# Polypharmacy and oral antidiabetic treatment for type 2 diabetes characterised by drug class and patient characteristics: A Japanese database analysis

**DOI:** 10.1038/s41598-019-49424-2

**Published:** 2019-09-10

**Authors:** Takeshi Horii, Makiko Iwasawa, Yusuke Kabeya, Koichiro Atuda

**Affiliations:** 1 Division of Clinical Pharmacy (Laboratory of Pharmacy Practice and Science I) and Research and Education Center for Clinical Pharmacy, Kitasato University School of Pharmacy, Sagamihara, Kanagawa Japan; 2 Division of Clinical Pharmacy (Laboratory of Drug Information) and Research and Education Center for Clinical Pharmacy, Kitasato University School of Pharmacy, Sagamihara, Kanagawa Japan; 3 Sowa Hospital, Department of Home Care Medicine, Sagamihara, Kanagawa, Japan

**Keywords:** Diabetes complications, Drug regulation

## Abstract

Polypharmacy (PP) occurs in patients with type 2 diabetes (T2DM) owing to multimorbidity. We evaluated concomitant PP and medication adherence in T2DM 3 years after initiation of administration of a hypoglycaemic agent using a nationwide claim-based database in Japan. Factors associated with medication PP and imperfect adherence were identified using multivariable logistic regression. PP was defined as using ≥6 medications. Patients with proportion of days covered (PDC) of <80% were defined as having poor medication adherence. A total of 884 patients were analysed. Multivariate analysis revealed that age, total number of consultations and body mass index (BMI) are factors that influence PP. Factors associated with PDC < 80% were 2–3, 4–5 and ≥ 6 medications compared with 1 medication, male sex, <17 consultations and age 50–59 and ≥ 60 years compared with <40 years. In conclusion, older age, high total number of consultations and BMI ≥ 25 kg/m^2^ are risk factors for PP. PP influenced good medication adherence at the end of the observation period.

## Introduction

In Japan, the number of patients with diabetes is steadily increasing; the National Health and Nutrition Examination Survey Japan 2018 estimated that 10 million people had diabetes^1^. Multiple medications are commonly prescribed for type 2 diabetes (T2DM). Management of hyperglycaemia, microvascular complications, macrovascular complications and medication side effects contribute to an increased number of medications among T2DM. Management of diabetes and its complications may improve target outcome but also contribute to the addition of unnecessary medications to the drug regimen^2^. Moreover, T2DM may seek medication therapy for diseases other than diabetes, such as hypertension or hyperlipidaemia, which can further contribute to the presence of polypharmacy (PP).

PP is associated with an increased risk for drug interactions, adverse events^3^, non-adherence and negative effects on activities of daily living and quality of life (QOL)^3,4^. Among hospitalised patients with T2DM in Japan, 64.6% report using 6 or more medications^5^. In the United States, 54% of T2DM cases were found to be associated with PP^6^. Older adults with diabetes are at a greater risk of receiving PP than those without diabetes^7^. Adherence to hypoglycaemic medications is important for successful T2DM care and patient outcomes related to comorbidity management. However, it is difficult to manage diabetes treatment over a long term. A study conducted in the United States reported that medication adherence was low in asymptomatic patients with diabetes^8^ and that 32% of patients with T2DM failed to continue treatment^9^. In a previous study in Japan, poor medication adherence was shown to increase the risk of diabetic microangiopathy^10^.

Many factors affect adherence to T2DM medication, such as socio-economic factors, healthcare providers, depression, age and gender^11^. However, factors related to medication adherence in drug-naïve patients with T2DM have not been evaluated in Japan. Here we evaluated the use of oral hypoglycaemic medications and adherence to PP and medication, including risks for PP and imperfect adherence in drug-naïve patients with T2DM, using a large-scale receipt database.

## Results and Discussion

**Figure 1:**
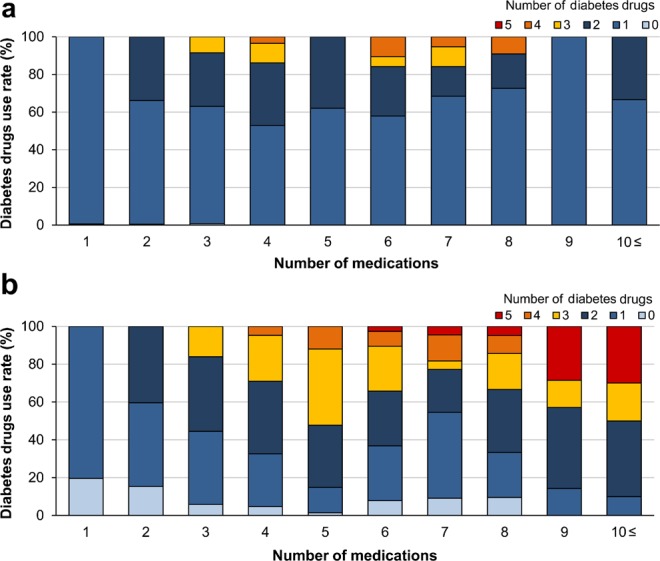
Number of medications and proportion of diabetes drugs. The number of medications and diabetes drugs at the start (**a**) and end (**b**) of the observation period are shown. At the start of the observation period, many diabetes drugs were used, whereas at the end of the observation period, the use of combination therapy showed an increasing trend.

The patients’ background characteristics are presented in Table 1. A total of 884 patients were analysed. The mean patient age was 47.0 ± 8.1 years, and 90.2% patients were male. At the start of the observation period, the number of internal medications was 2.4 ± 1.8 and the rate of PP, defined ≥6 internal medications, was 6.7%. DPP-4 inhibitors were the most commonly used hypoglycaemic medications, followed by biguanides, α-glucosidase inhibitors and sulfonylureas. The number of medications and proportion of diabetes drugs are presented in Fig. 1. At the end of the observation period, the frequency of the use of diabetes drugs and combination therapy tended to increase compared with those at the start of the observation period. Multivariate analysis with PP as the objective variable (Table 2) revealed statistically significant difference for age 40–49 years (OR: 2.71, 95% CI: 1.02–7.22, p = 0.046), 50–59 years (OR: 2.96, 95% CI: 1.10–7.99, p = 0.032) and ≥60 years (OR: 4.43, 95% CI: 1.31–15.0, p = 0.017) compared with that for age <40 years; for total number of consultations during the 3-year observation period of 11–20 times (OR: 5.56, 95% CI: 1.56–20.0, p = 0.008), 21–30 times (OR: 9.63, 95% CI: 2.82–32.8, p < 0.001) and ≥30 times (OR: 11.6, 95% CI: 3.52–38.5, p < 0.001) compared with 1–10 times and for body mass index (BMI) ≥25 kg/m^2^ (OR: 1.71, 95% CI: 1.04–2.80, p < 0.033).

**Table 1:** Patients’ background characteristics.

	Total cases (n = 884)
Number of medications	2.4 ± 1.8
Male (%)	797 (90.2)
Age (years)	47.0 ± 8.1
Polypharmacy, n (%)	59 (6.7)
Smoking, n (%)	331 (37.4)
**Drinking frequency**, **n (%)**	
Every day	180 (20.4)
Occasionally	214 (24.2)
Scarcely	321 (36.3)
Exercise habits, n (%)	128 (14.5)
HbA1c (%)	7.9 ± 2.0
BMI (kg/m^2^)	26.5 ± 4.7
**Diabetes drug use rate**, **n (%)**	
DPP-4 inhibitor	301 (34.0)
αGI	208 (23.5)
Glinide	38 (4.3)
SU drug	204 (23.1)
Biguanide	235 (26.6)
Thiazolidine	130 (14.7)
SGLT-2 inhibitor	0 (0)

HbA1c, haemoglobin A1c; BMI, body mass index; DPP-4 inhibitor, dipeptidyl peptidase-4 inhibitor; αGI, α-glucosidase inhibitor; SU, sulfonylurea; SGLT2 inhibitor, Sodium glucose cotransporter 2 inhibitor.

**Table 2:** Factors associated with polypharmacy at the end of the observation period, multiple logistic regression model.

	Odds ratio	95% CI	P-value
Sex: Male	2.63	0.91–7.56	0.073
**Age (years)**			
<40 (n = 144)	Reference		
40–49 (n = 387)	2.71	1.02–7.22	0.046
50–59 (n = 301)	2.96	1.10–7.99	0.032
≥ 60 (n = 52)	4.43	1.31–15.0	0.017
**Number of consultations in 3 years**			
1–10 (n = 207)	Reference		
11–20 (n = 174)	5.56	1.56–20.0	0.008
21–30 (n = 196)	9.63	2.82–32.8	<0.001
≥30 (n = 307)	11.6	3.52–38.5	<0.001
BMI (kg/m^2^) ≥ 25	1.71	1.04–2.80	0.033
SU drug	1.32	0.78–2.25	0.309

BMI, body mass index; SU, sulfonylurea.

The median PDC was 79.6% (31.7–96.1), and 50.2% of the patients showed poor medication adherence. Receiver operating characteristic (ROC) analysis for the total number of consultations for PDC < 80% revealed that the cut-off value was 17 times during the 3 years (area under the curve: 0.86, sensitivity: 95.9%, specificity: 60.6%). Multivariate analysis with PDC < 80% as the objective variable (Table 3) revealed that statistical significance was observed for 2–3 medications (OR: 0.34, 95% CI: 0.22–0.51, p < 0.001), 4–5 medications (OR: 0.13, 95% CI: 0.08–0.23, p < 0.001) and ≥6 medications (OR: 0.18, 95% CI: 0.10–0.34, p < 0.001) compared with 1 medication at the end of the observation; male sex (OR: 2.20, 95% CI: 1.18–4.08, p = 0.013); total number of consultations <17 times (OR: 27.1, 95% CI: 15.6–47.0, p < 0.001) and age 50– < 60 years (OR: 0.39, 95% CI: 0.23–0.68, p < 0.001) and ≥60 years (OR: 0.37, 95% CI: 0.15–0.89, p = 0.026) compared with <40 years.

**Table 3:** Factors influencing poor medication adherence, multiple logistic regression model.

	Odds ratio	95% CI	P-value
**Number of medications at the end**			
0.1 (n = 372)	Reference		
2.3 (n = 261)	0.34	0.22–0.51	<0.001
4.5 (n = 153)	0.13	0.08–0.23	<0.001
≥6 (n = 98)	0.18	0.10–0.34	<0.001
Sex: Male	2.20	1.18–4.08	0.013
**Age (years)**			
<40 (n = 144)	Reference		
40–49 (n = 387)	0.74	0.44–1.23	0.245
50–59 (n = 301)	0.39	0.23–0.68	0.001
≥60 (n = 52)	0.37	0.15–0.89	0.026
**Total number of consultations/3** **yrs**.**(times)**			
≥17 (n = 597)	Reference		
<17 (n = 287)	27.1	15.6–47.0	<0.001
DPP-4 inhibitor	0.73	0.49–1.10	0.132
Biguanide	0.62	0.40–0.96	0.512
Thiazolidine	1.08	0.63–1.86	0.781

DPP-4 inhibitor, dipeptidyl peptidase-4 inhibitor.

The present study revealed that the risk factors for PP were older age, high total number of consultations and BMI ≥ 25 kg/m^2^. The rate of PP occurrence in the present study was 6.7%, much lower than the approximately 50% rate previously reported^12^. The low rate may be attributed to our patients being younger (47.0 ± 8.1 years) and the presence of fewer comorbidities owing to the exclusion of patients with a history of prescription for hypoglycaemic medications. In the presence or absence of diabetes, the prevalence of hypertension and dyslipidaemia increases with age^11,13^. In addition, the risk of T2DM onset increases during the middle years of life^14^; therefore, the duration of diabetes becomes longer as age increases. Simultaneously, the incidence of microangiopathy and its related disorders also increases^15,16^, thereby causing an increase in the risk of PP. Patients with a high total number of consultations are likely to have poor medical conditions and multiple complications. Thus, such patients may become polypharmic; however, further investigation is necessary to confirm this aspect. In overweight diabetic patients (BMI ≥ 25 kg/m^2^), the accumulation of visceral fat is a known risk factor for cardiovascular events^17^; these events adversely affect blood pressure, lipid metabolism and blood glucose control. Risk of PP increases as drug therapies according to guidelines are initiated for each disease.

Next, we investigated the factors associated with poor medication adherence and found that OR was the lowest in the cohort with 4–5 internal medications and that ORs with ≥6 medications were only slightly increased. Several studies reported that T2DM with a high number of medications^18^ and complications^19–22^ had a positive association with adherence. These reports could partially explain why participants with 4–5 medications in the present study had a lower barrier for medication. On the other hand, medication adherence assumedly decreases as the number of internal medications increases and usage becomes more complicated; therefore, we believe that OR for good medication adherence can be achieved with ≥6 medications, which is marginally higher compared with 4–5 medications.

In accordance with previous studies^23–25^, there was a distinct trend for increased adherence with increasing age in the present study. Male sex was a risk factor for poor medication adherence in the present study; nevertheless, the relationship between sex and medication adherence remains to be established. Males were reported to have a higher medication adherence than females^23^; however, several studies reported^24–26^ no sex-related differences in medication adherence. Analysis of the relationship between medication adherence and total number of consultations during the observation period resulted in a cut-off value of 17 times. Multivariate revealed found that <17 consultations during the observation period was a risk factor for poor medication adherence. Optimisation of prescriptions considering the patient’s condition and the decreased frequency of guidance to medication adherence by pharmacists may have led to a lower frequency of consultations. Furthermore, it is possible that missed consultations or treatment interruptions are reflected in the lower frequency of consultation; therefore, this became a risk factor for poor medication adherence. Differences in medication adherence were expected depending on the class of the drugs because the methods of taking hypoglycaemic medications are diverse (e.g. once a day after meals or thrice a day immediately before every meal); however, there was no such differences were observed.

This study had several limitations. First, this was a retrospective study conducted using a large-scale receipt data; therefore, information and selection biases, may have affected the results. Second, owing to the small number of patients aged ≥ 65 years, it was difficult to examine whether this age range was a risk factor for PP. Third, as most patients were male, further analyses are required to determine whether these findings apply to females as well. Finally, in this study, whether the medication was administered to inpatients or outpatients could not be distinguished. Medication management during hospitalisation may be performed by a medical practitioner, which may affect the outcomes of PDC.

Risk of PP is high in elderly patients and in patients with a high BMI. To avoid PP in such patients, evaluation and implementation of an appropriate drug therapy are necessary. Furthermore, higher numbers of consultations over a certain period are associated with a higher risk of PP. The good medication adherence observed in this study suggests that the prescriptions were optimised such that good medication adherence was possible even with PP. It is necessary to consider just how well patient prescriptions are optimised in clinical practice to determine if there is room for further improvement via intervention. The efficacy of telemedicine, such as online medical treatment to avoid increasing patient burden from frequent visits, should also be examined. Finally, although PP was speculated to be a risk factor for poor medication adherence, the contrary was observed in this study.

## Methods

This retrospective, cohort study was conducted using large-scale receipt data, which houses a database of inpatient, outpatient and pharmacy claims from enrollees of employer-sponsored health plans throughout all prefectures in Japan^27,28^. Medical receipts contain information, such as disease code [Medical Information System Development Center standard disease master and International Classification of Diseases-10 (ICD-10)], prescription details and medical practices in addition to the patients’ basic details. Patients were registered between May 2005 and January 2013. The inclusion criteria were as follows: (1) T2DM (ICD-10 code: E11) or diabetes (ICD-10 code: E14), (2) prescription history of hypoglycaemic medication and (3) capable of continuous follow-up observation for 3 years. The exclusion criteria were as follows: (1) type 1 diabetes, (2) age <18 years or ≥75 years or (3) insulin and glucagon-like peptide-1 agonist use. Index date was defined as the start month of the prescribed diabetes medication.

### Definition of polypharmacy

Of all medications, injections, external medicine, internal medicine with consistent usage and antibiotics to be used for <7 consecutive days were excluded. Although the number of medications has not been not strictly defined for PP, it was defined as ≥6 internal medications for this study in accordance with previous reports in Japan. The number of internal medicines was defined as the number of drugs prescribed at the time of the completion of observations^29^.

### Definition of medication adherence

Proportion of days covered (PDC) was calculated for each patient as an index of medication adherence. PDC was defined as the proportion of days for which the patient was prescribed and actually possessed hypoglycaemic medications during the observation period divided by the observation period. The formula used is as follows:

$${\rm{PDC}}( \% )=[{\rm{number}}\,{\rm{of}}\,{\rm{days}}\,{\rm{possessing}}\,{\rm{hypoglycaemic}}\,{\rm{medications}}\,{\rm{during}}\,{\rm{the}}\,{\rm{observation}}\,{\rm{period}}/{\rm{observation}}\,{\rm{period}}({\rm{days}})]\times 100$$

**Figure 2:**
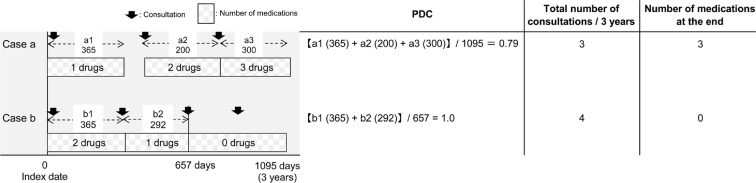
Proportion of days covered (PDC) based on the total number of consultations in 3 years and the number of medications at the end of the observation period. Case a: The prescription days of a1, a2 and a3 were added, and PDC was determined by dividing the number by 1095 days of the observation period. The number of drugs at the end of observation period corresponds to the number of drugs prescribed in a3. Case b: Since the medication use was completed at the third (b2) and subsequent consultations, the observation period for medication adherence was 657 days.The prescription days of b1 and b2 were added, and PDC was determined by dividing the number by 657 days of the observation period. The number of drugs at the end of observation period corresponds to the number of drugs prescribed in b3. Thus, the number of drugs at the end of the observation period is 0.

PDC was calculated based on the total number of consultations in 3 years and the number of medications at the end of the observation period (Fig. 2). Patients with PDC < 80% were defined as having poor medication adherence. Data on age and sex were collected using receipt information from the same year as that of the index date used to obtain the baseline patient backgrounds. Data on BMI were collected using receipt information from the same year as that of the observation start month. Haemoglobin A1c (HbA1c) data were collected from the receipt information of the same year as that of the observation start month and the receipt information 3 years after the observation start month. The total number of consultations for diabetes treatment during the 3-year observation period (total number of consultations) and the number of consultation days on which hypoglycaemic drugs were prescribed during the observation period were counted. Information on smoking and alcohol consumption frequencies was obtained from the results of the questionnaire administered to the patients at the time of medical examination in the same year as that of the start of observation.

### Statistical analysis

Patient data were expressed as mean ± S.D. With PDC < 80% as the objective variable, receiver-ROC analysis was conducted to calculate a cut-off value for the total number of consultations. Factors related to PP at the end of observation were examined using logistic regression analysis. Sex, age at baseline (<40, 40–49, 50–59 and ≥ 60 years), HbA1c with a cut-off value of 7.0%, BMI with a cut-off value of 25 kg/m^2^, presence or absence of smoking, drinking frequency, presence or absence of exercise habits, use of hypoglycaemic agents for each medication and total number of consultations with the cut-off value calculated using ROC analysis were used as explanatory variables. Factors related to poor medication adherence were examined by adding the number of internal medications (0–1, 2–3, 4–5 or ≥6) to these explanatory variables at the end of the observation period. Based on the blood glucose control target values of diabetic patients in Japan, 7.0% was set as the cut-off value for HbA1c and 25 kg/m^2^, which is the target value for weight control, was set as the cut-off value for BMI. Univariate analysis was conducted for each explanatory variable. We performed univariate analysis on each of these predictor variables and multivariate analysis using factors for which P < 0.2 and calculated the odds ratio. Statistical analysis was performed using Stata10, and P < 0.05 was considered statistically significant.

### Ethical considerations

Because we used unlinkable, anonymised data, this study did not require adherence to the Ethical Guidelines for Medical and Health Research Involving Human Subjects and the approval of the institutional review board committee of the Kitasato University and was, therefore, exempted from institutional review board consideration.

## Data Availability

The datasets analysed during the current study are available from the corresponding author on reasonable request.
